# Efficacy and safety of triple *versus* dual antithrombotic therapy in atrial fibrillation and ischemic heart disease: a systematic review and meta-analysis

**DOI:** 10.18632/oncotarget.20870

**Published:** 2017-09-14

**Authors:** Wengen Zhu, Linjuan Guo, Fadi Liu, Rong Wan, Yang Shen, Gregory Y.H. Lip, Kui Hong

**Affiliations:** ^1^ Department of Cardiovascular Medicine, the Second Affiliated Hospital of Nanchang University, Nanchang of Jiangxi, China; ^2^ Jiangxi Key Laboratory of Molecular Medicine, Nanchang of Jiangxi, China; ^3^ University of Birmingham Centre for Cardiovascular Sciences, City Hospital, Birmingham, United Kingdom

**Keywords:** atrial fibrillation, ischemic heart disease, triple therapy, dual therapy

## Abstract

The optimal antithrombotic regimen for patients with atrial fibrillation and ischemic heart disease remains unclear. Therefore, we aimed to compare the efficacy and safety of triple therapy (TT [an anticoagulant and 2 antiplatelet drugs]) with dual therapy (DAPT [2 antiplatelet drugs] or DT [an anticoagulant and a single antiplatelet drug]) in patients with atrial fibrillation and ischemic heart disease. We systematically searched the Cochrane Library, PubMed and Embase databases for all relevant studies up to August 2017. The overall risk estimates were calculated using the random-effects model. A total of 17 observational studies were included. Regarding the efficacy outcomes, no differences were observed between the triple therapy and the dual therapy for all-cause death, cardiovascular death, or thrombotic complications (i.e., acute coronary syndrome, stent thrombosis, thromboembolism/stroke, and major adverse cardiac and cerebrovascular events). Regarding the safety outcomes, compared with DAPT, TT was associated with increased risks of major bleeding (a relative risk of 1.96 [1.40–2.74]), minor bleeding (1.69 [1.06–2.71]) and overall bleeding (1.80 [1.23–2.64]). Compared wtih DT, TT was associated with a greater risk of major bleeding (1.65 [1.23–2.21]), but rates of minor bleeding (0.99 [0.56–1.77]) and overall bleeding (1.14 [0.76–1.71]) were similar. Overall, TT confers an increased hazard of major bleeding with no thromboembolic protection compared with dual therapy in patients with atrial fibrillation and ischemic heart disease.

## INTRODUCTION

Approximately 20–30% of atrial fibrillation (AF) patients have coexisting ischemic heart disease (IHD) [1]. Both AF and IHD confer an increased risk of thrombotic complications [2]. Oral anticoagulation (OAC) agents and antiplatelet therapies are beneficial for AF and IHD patients, respectively [1]. However, selecting the optimal antithrombotic therapy for patients with both AF and IHD remains a challenge, especially for those patients with a high risk of thrombotic complications and bleeding [3]. Recently, 3 antithrombotic strategies are used in the management of patients with IHD and AF: triple therapy (TT [an anticoagulant plus 2 antiplatelet drugs]) and two types of dual therapy (DAPT [2 antiplatelet drugs] or DT [an anticoagulant plus a single antiplatelet drug]). However, the evidence related to these antithrombotic strategies has yielded conflicting results. Some of the studies have reported a decreased risk of thrombotic complications in patients on TT [4, 5], whereas other studies have demonstrated that the high risk of bleeding associated with TT might outweigh its benefits [6–8]. Dual antithrombotic therapy was found to decrease the risk of major adverse cardiac events in a large study of patients with AF and IHD [9] but did not decrease the risk of coronary death or myocardial infarction (MI) in another study [10]. In this meta-analysis, we aimed to compare the efficacy and safety of TT with those of dual therapies (DAPT or DT) in patients with AF and IHD.

## MATERIALS AND METHODS

We conducted this study according to the meta-analysis of observational studies in epidemiology (MOOSE) guidelines [11] and the Preferred Reporting Items for Systematic Reviews and Meta-Analyses (PRISMA) guidelines [12].

### Data sources and searches

We systematically searched the Cochrane Library, PubMed and Embase databases for studies comparing the efficacy and safety of triple therapy with dual therapy in patients with AF and IHD from database inception until August 2017. To identify studies involving antithrombotic therapies, we used the following keywords: *‘dual therapy’, ‘triple therapy’, ‘antiplatelet’, ‘antithrombotic’, ‘anticoagulant’, ‘aspirin’, ‘warfarin’, ‘vitamin K antagonists’, ‘acenocoumarol’, ‘phenprocoumon’, ‘thienopyridine’, ‘clopidogrel’, ‘prasugrel’, ‘cilostazol’ and ‘ticlopidine’*. To identify studies involving relevant participants, we used the following keywords: *‘atrial fibrillation’, ‘ischemic heart disease’, ‘coronary heart disease’, ‘acute coronary syndromes’, ‘angina pectoris’, and ‘coronary stenting’*. To identify studies involving relevant outcomes, we used the following keywords: *‘all-cause mortality’, ‘cardiovascular mortality’, ‘mortality’, ‘death’, ‘myocardial infarction’, ‘stroke’, ‘thromboembolism’, ‘cerebrovascular accident’, ‘major adverse cardiac and cerebrovascular events’, ‘major bleeding’, ‘minor bleeding’, ‘bleeding’ and ‘hemorrhage’*. These 3 categories of keywords were combined using the Boolean operator “and”. A detailed description of the electronic search strategies is provided in Supplementary Table 1. The references lists of the included studies were also searched, and we did not impose language restrictions on our searches.

### Study selection criteria

Studies were included if they met the following criteria: (a) compared the safety and efficacy of triple therapy with dual therapy; (b) included patients with nonvavular AF and IHD; and (c) reported the efficacy outcomes, including all-cause death, cardiovascular death, acute coronary syndrome (unstable angina and nonfatal MI), stent thrombosis, thromboembolism(TE)/stroke, and major adverse cardiac and cerebral events (MACCEs) or reported on the safety outcomes, including major bleeding, minor bleeding and overall bleeding. The definitions of outcomes adopted by the original studies are summarized in Supplementary Table 2. To avoid underpowered data, we excluded studies with follow-up durations of less than 1 year. In cases of duplicate publications, we included the publication with the longest follow-up duration or the largest number of study participants. Studies with insufficient data were also excluded (*e.g.,* certain publication types with no statistics [*e.g.,* reviews, letters, and case reports], and clinical studies that did not report the risk estimates or relevant outcomes).

### Patient involvement

No patients were involved in setting the research question, in the outcome measures, in the design, or in the implementation of the study. No patients were asked for advice on the interpretation or writing up of the results. There are no plans to disseminate the results of the research to study participants or to the relevant patient community.

### Data extraction and quality assessment

Three independent reviewers (W.G.Z., L.J.G. and F.D.L.) screened all of the identified titles and/or abstracts and then retrieved the full-texts of the shortlisted studies. Disagreements were resolved via discussion with a fourth reviewer (K.H.). The data were extracted from each included study as follows: the first author, year of publication, duration of follow-up, ages at baseline, sample size, outcomes, proportion of female participants, number of participants receiving each treatment regimen, data source, antithrombotic medications, and relative risks [RRs] with the 95% confidence intervals [CIs]. If both unadjusted and adjusted RRs were available in one study, the most adjusted RRs were extracted. Three reviewers (W.G.Z., L.J.G. and F.D.L.) independently assessed the included studies’ qualities using the Newcastle-Ottawa quality assessment scale (NOS) [13]. NOS scores < 6 indicated low quality studies, and scores ≥ 6 indicated moderate-high quality studies.

### Statistical analyses

We performed all of the statistical analyses using the Review Manager 5.3 software (Cochrane Collaboration, Copenhagen, Denmark). The efficacy and safety outcomes were defined dichotomously, and we compared their occurrence risks between triple therapy and dual therapy. The statistical analyses were performed as previously described [14]. In brief, the RRs were used as the common risk estimates, and we calculated the natural logarithm of the RR (log[RR]) and its standard error (SE_log[RR]_) for each study. In the consistency test, the heterogeneity was assessed with the *I*^2^ statistical test for which *I*^2^ values < 25%, 25–50%, and > 75% were considered indicative of low, moderate, and high levels of heterogeneity, respectively. Owing to the heterogeneity inherent (both clinically and methodologically) to the included studies, the log[RR] and SE_log[RR]_ values were pooled with the random-effects model, which is a more conservative method than the fixed-effects model [15]. We determined the degree of possible publication bias by inspecting funnel plots. To evaluate the influence of individual studies on the pooled data, we conducted sensitivity analyses by removing the included studies one by one. A *P*-value less than 0.05 indicated statistical significance.

## RESULTS

### Study selection

**Figure 1:**
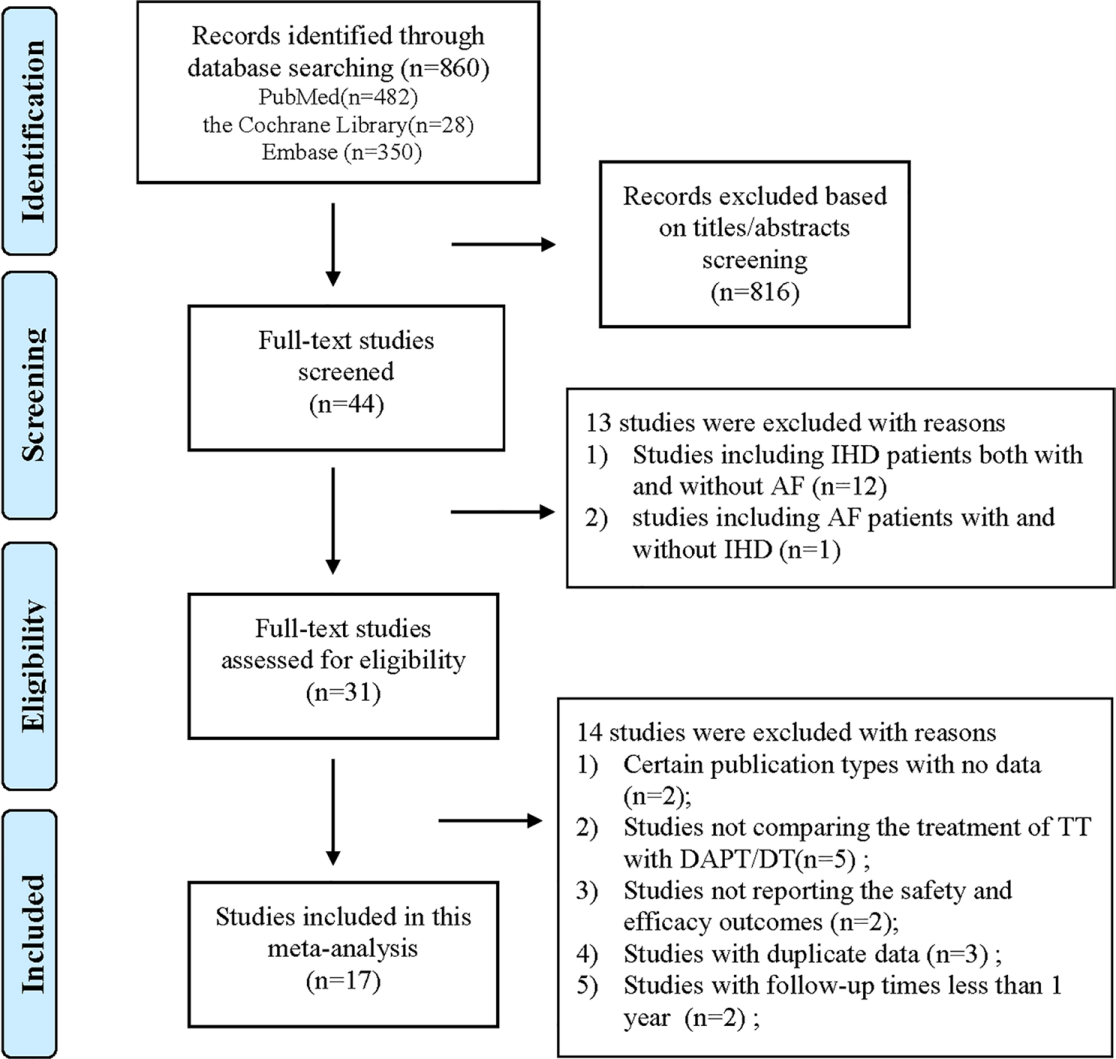
Flow chart of the study selection process for this meta-analysis Abbreviations: IHD = ischemic heart disease; AF = atrial fibrillation; TT = triple therapy (an oral anticoagulant plus 2 antiplatelet drugs); DAPT = dual therapy (2 antiplatelet drugs); DT = dual therapy (an oral anticoagulant plus one antiplatelet drug).

Figure 1 presents a flow chart of the study selection process. We identified 860 relevant studies (28 through the Cochrane Library, 482 through PubMed, and 350 through Embase). No additional studies were identified through manual searches. A total of 816 studies were excluded based on their titles or abstracts. The remaining 44 studies were eligible for detailed full-text evaluations. Twenty-seven of those studies were excluded for the following reasons: (1) they were studies that included IHD patients both with and without AF (*n* = 12) [16–27] or studies including AF patients with and without IHD (*n* = 1) [28]; and (2) the studies had insufficient data (*n* = 9; 5 studies did not compare the outcomes of triple therapy with those of dual therapy [29–33], 2 studies did not report the outcomes of interest [34, 35], and 2 studies did not report the risk estimates [36, 37]), duplicate data (*n* = 3) [38–40] or follow up data of less than 1 year (*n* = 2) [41, 42]. Ultimately, 17 studies [4–10, 43–52] (8 prospective and 9 retrospective studies) were included in this meta-analysis. The baseline characteristics of these included studies are presented in Supplementary Table 4.

### Quality assessment and publication bias

As illustrated in Supplementary Table 3, all of the included studies had an NOS score ≥ 6 (graded as moderate to high quality). As shown in Supplementary Figure 1, visual inspection of the funnel plots of the efficacy and safety outcomes between the triple therapy and dual therapy indicated no major publication bias.

### Efficacy outcomes of the triple *versus* the dual therapies

**Figure 2:**
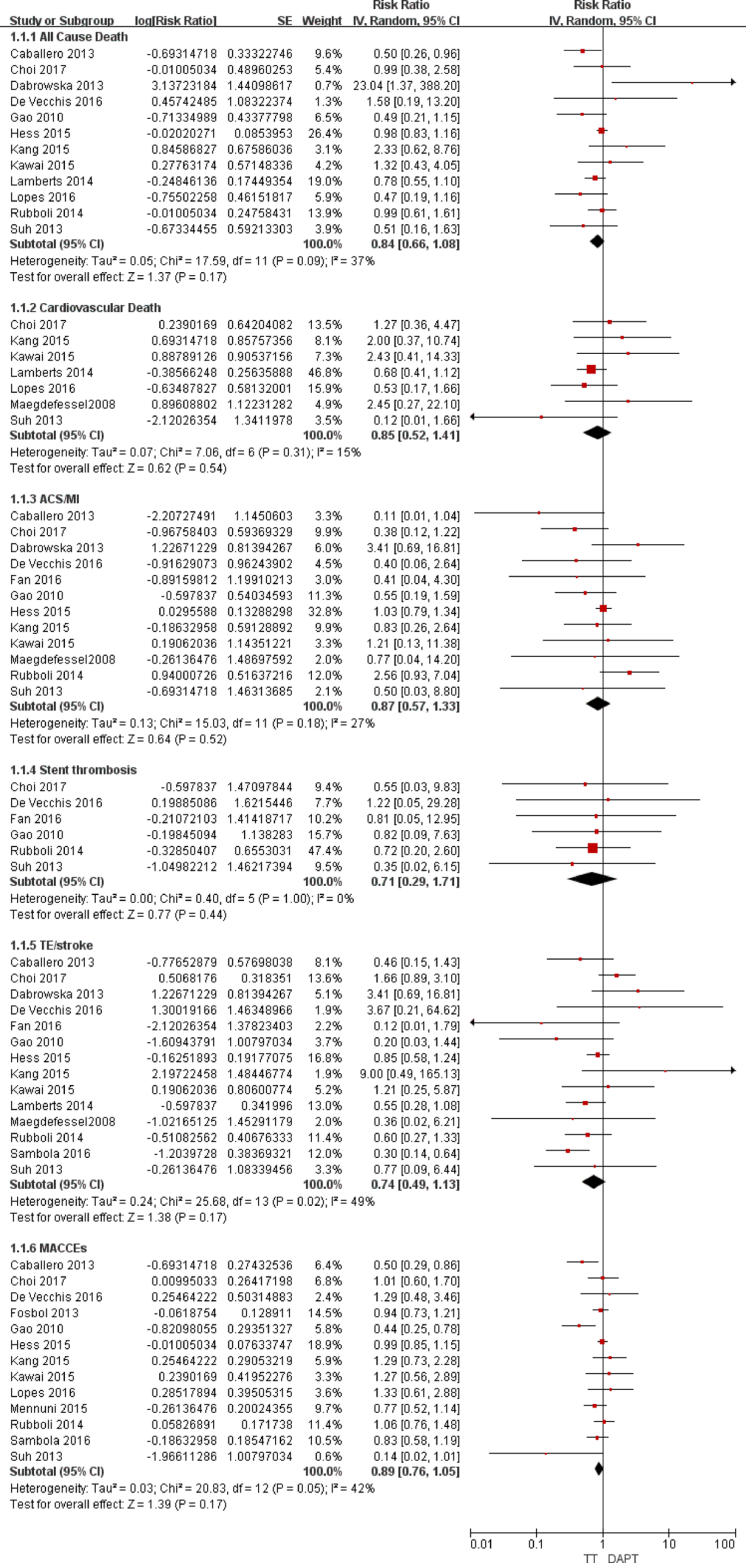
Forest plot for the comparative analysis of the efficacies of TT and DAPT in patients with AF and IHD Abbreviations: IHD = ischemic heart disease; AF = atrial fibrillation; TT = triple therapy (an oral anticoagulant plus 2 antiplatelet drugs); DAPT = dual therapy (2 antiplatelet drugs); MI = myocardial infarction; ACS = acute coronary syndrome; TE = thromboembolism; MACCEs = major adverse cardiac and cerebrovascular events; SE = standard error; CI = confidence interval; IV = inverse of the variance.

**Figure 3:**
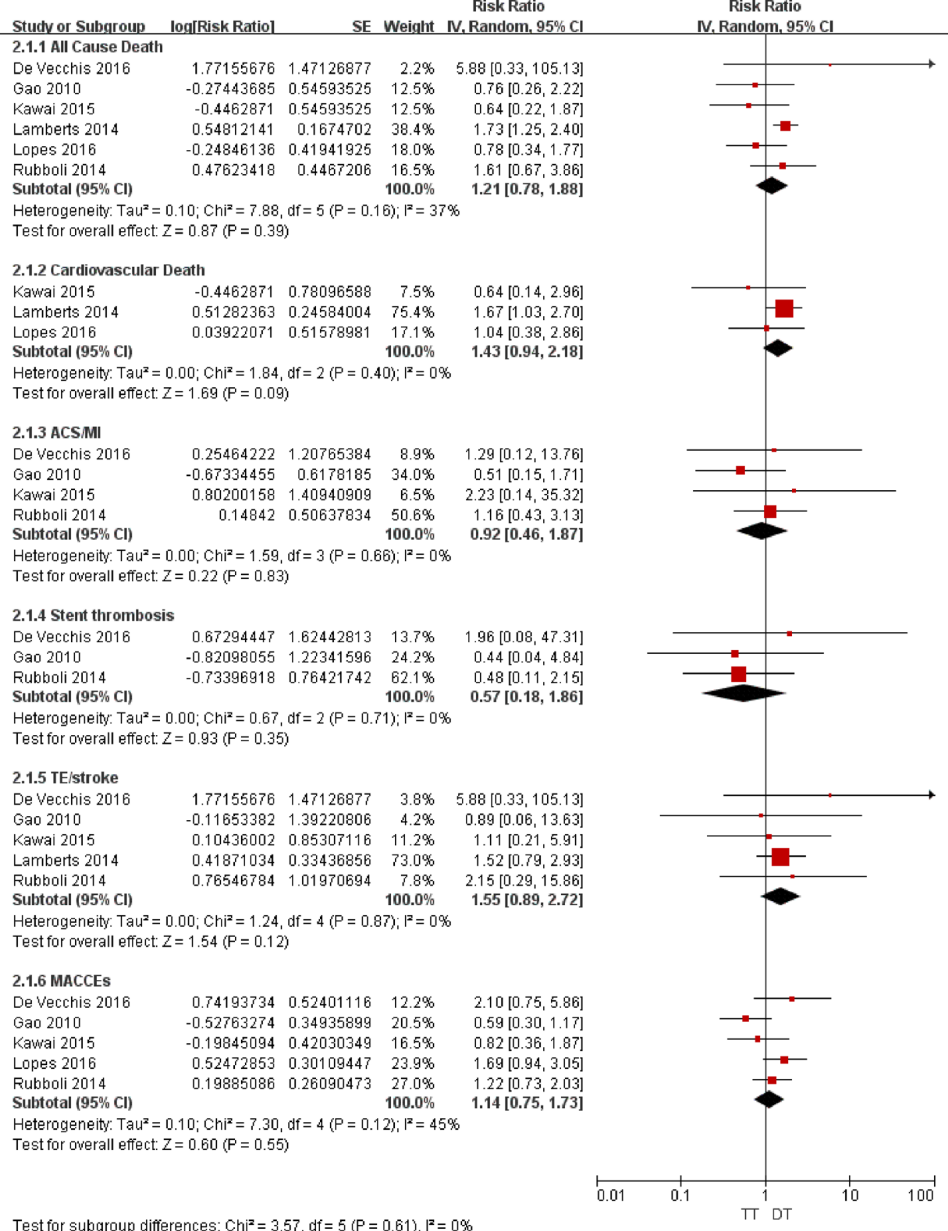
Forest plot for the comparative analysis of the efficacies of TT and DT in patients with AF and IHD Abbreviations: IHD = ischemic heart disease; AF = atrial fibrillation; TT = triple therapy (an oral anticoagulant plus 2 antiplatelet drugs); DT = dual therapy (an oral anticoagulant plus one antiplatelet drug); MI = myocardial infarction; ACS = acute coronary syndrome; TE = thromboembolism; MACCEs = major adverse cardiac and cerebrovascular events; SE = standard error; CI = confidence interval; IV = inverse of the variance.

When comparing the efficacy outcomes between the triple therapy and the dual therapy, we focused on the outcomes of all-cause death, cardiovascular death and thrombotic complications (acute coronary syndrome, stent thrombosis, TE/stroke and MACCEs). As presented in Figures 2 and 3, the consistency test indicated a low to moderate degree of heterogeneity, i.e., the *I*^2^ values ranged from 0% to 49%.

### Association with death

For all-cause death, the random-effects model analysis indicated no difference in the risks between TT and DAPT (RR = 0.84; 95% CI: 0.66–1.08; *P* = 0.17; Figure 2) or between TT and DT (RR = 1.21; 95% CI: 0.78–1.88; *P* = 0.39; Figure 3). For cardiovascular death, the risks were comparable between TT and DAPT (RR = 0.85; 95% CI: 0.52–1.41; *P* = 0.54; Figure 2) and between TT and DT (RR = 1.43; 95% CI: 0.94–2.18; *P* = 0.09; Figure 3).

### Association with thrombotic complications

The ACS/MI risks were comparable between TT and DAPT (RR = 0.87; 95% CI: 0.57–1.33; *P* = 0.52; Figure 2) and between TT and DT (RR = 0.92; 95% CI: 0.46–1.87; *P* = 0.83; Figure 3). For stent thrombosis, the pooled analysis indicated no difference in the risks between TT and DAPT (RR = 0.71; 95% CI: 0.29–1.71; *P* = 0.44; Figure 2) or between TT and DT (RR = 0.57; 95% CI: 0.18–1.86; *P* = 0.35; Figure 3). For the outcome of TE/stroke, there was also no difference between TT and DAPT (RR = 0.74; 95% CI: 0.49–1.13; *P* = 0.17; Figure 2) or between TT and DT (RR = 1.55; 95% CI: 0.89–2.72; *P* = 0.12; Figure 3). Finally, the risk of MACCEs was comparable between TT and DAPT (RR = 0.89; 95% CI: 0.76–1.05; *P* = 0.17; Figure 2) and between TT and DT (RR = 1.14; 95% CI: 0.75–1.73; *P* = 0.55; Figure 3).

### Sensitivity analysis

None of the RR values changed substantially following the removal of the included studies one by one. For TT *versus* DAPT, the results were stable when we re-performed these analyses with fixed effects models.

### Safety outcomes of the triple *versus* the dual therapies

Major bleeding, minor bleeding, and overall bleeding were regarded as the safety outcomes. The consistency test indicated a moderate to high heterogeneity for TT *versus* DAPT (*I*^2^ ranging from 58% to 72%) and a low heterogeneity for TT *versus* DT (*I*^2^ ranging from 0% to 11%).

### Association with bleeding

**Figure 4:**
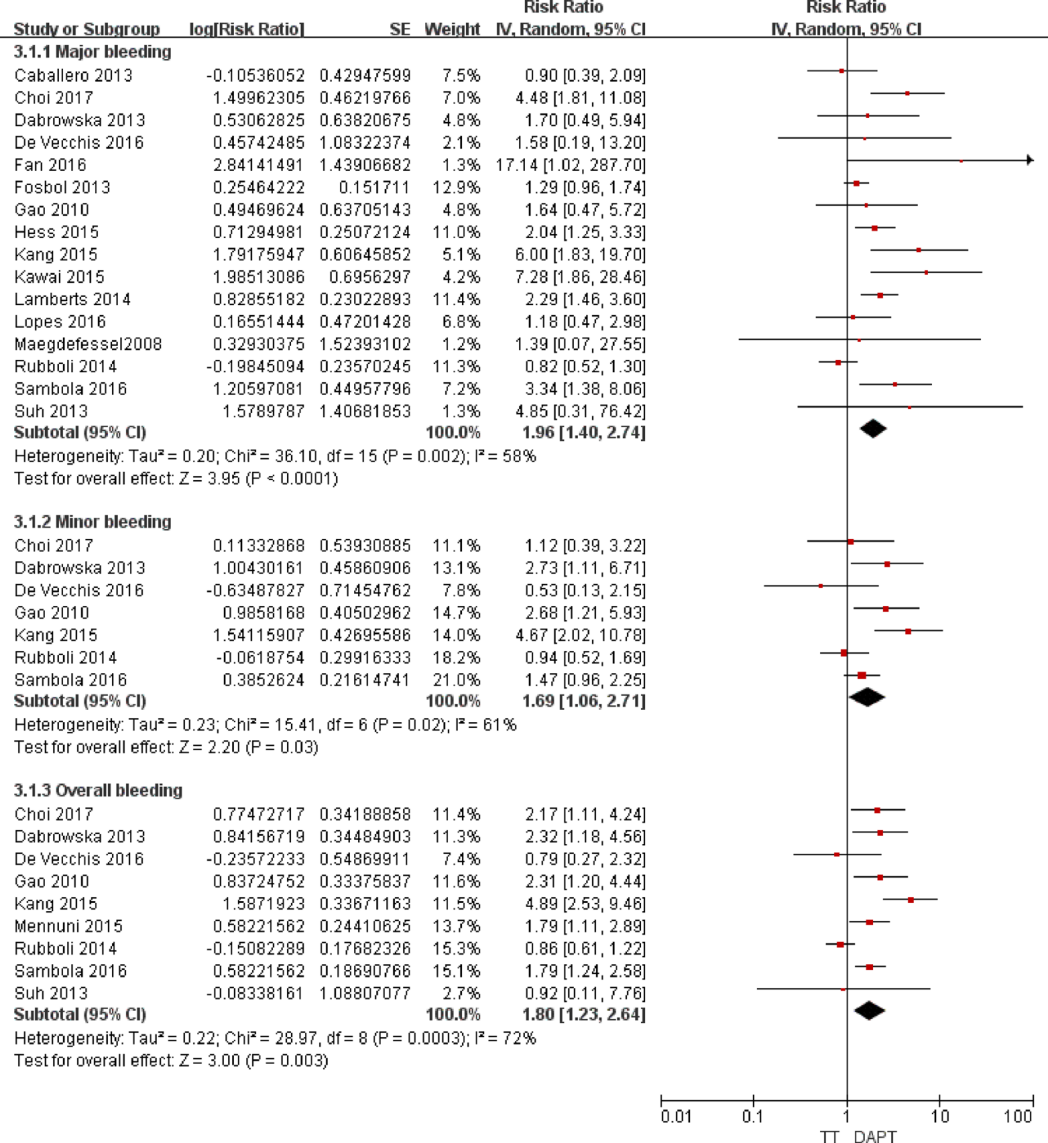
Forest plot for the comparative analysis of the safeties of TT and DAPT in patients with AF and IHD Abbreviations: IHD = ischemic heart disease; AF = atrial fibrillation; TT = triple therapy (an oral anticoagulant plus 2 antiplatelet drugs); DAPT = dual therapy (2 antiplatelet drugs); MI = myocardial infarction; MACCEs = major adverse cardiac and cerebrovascular events; SE = standard error; CI = confidence interval; IV = inverse of the variance.

**Figure 5:**
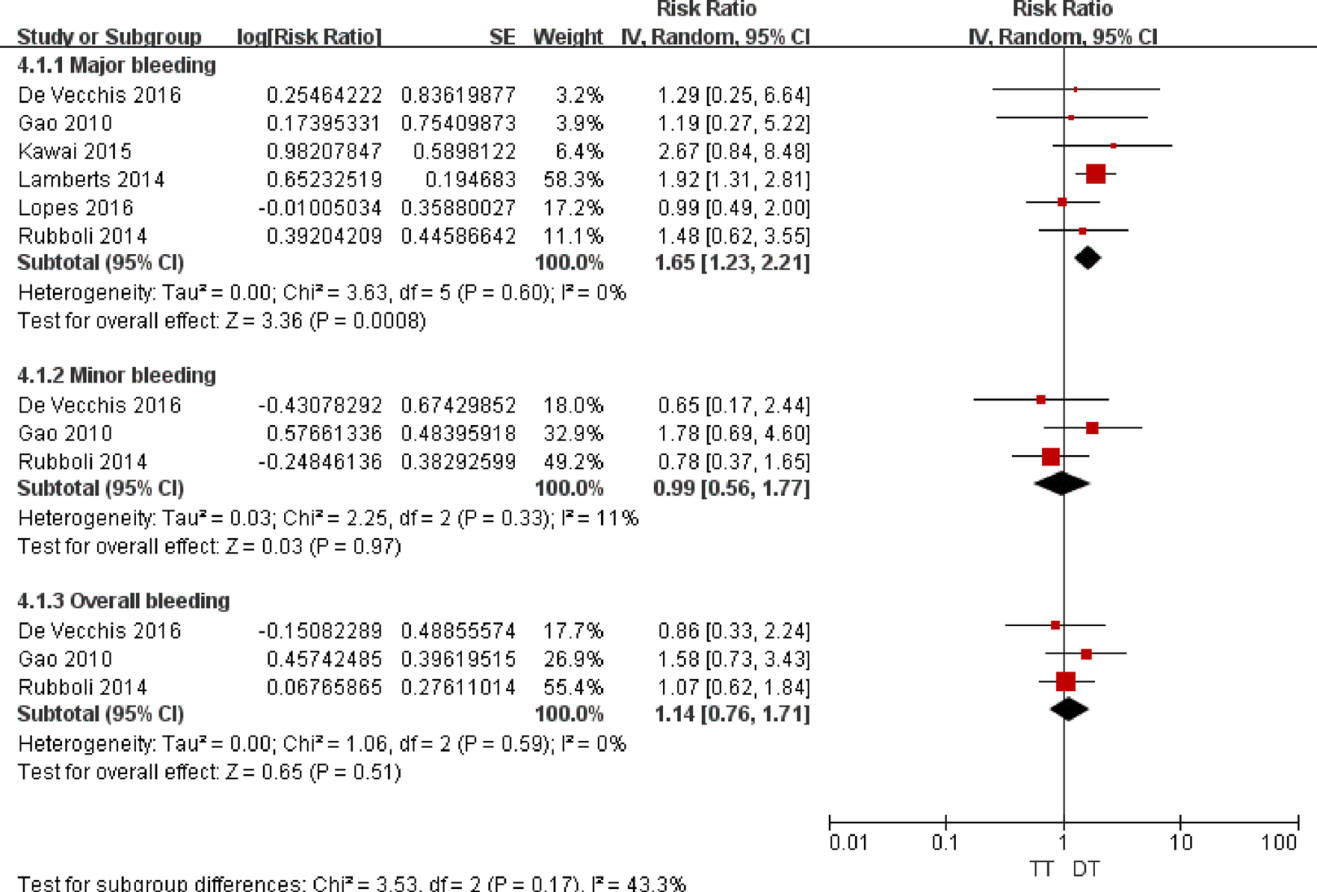
Forest plot for the comparative analysis of the safeties of TT and DT in patients with AF and IHD Abbreviations: IHD = ischemic heart disease; AF = atrial fibrillation; TT = triple therapy (an oral anticoagulant plus 2 antiplatelet drugs); DT = dual therapy (an oral anticoagulant plus one antiplatelet drug); MI = myocardial infarction; MACCEs = major adverse cardiac and cerebrovascular events; SE = standard error; CI = confidence interval; IV = inverse of the variance.

As illustrated in Figure 4, compared with DAPT, TT was associated with increased risks of major bleeding (RR = 1.96; 95% CI: 1.40–2.74; *P* < 0.0001), minor bleeding (RR = 1.69; 95% CI: 1.06–2.71; *P* = 0.03), and overall bleeding (RR = 1.80; 95% CI: 1.23–2.64; *P* = 0.03). As presented in Figure 5, compared with DT, TT was associated with an increased risk of major bleeding (RR = 1.65; 95% CI: 1.23–2.21; *P* = 0.0008) but with similar rates of minor bleeding (RR = 0.99; 95% CI: 0.56–1.77; *P* = 0.97) and overall bleeding (RR = 1.14; 95% CI: 0.76–1.71; *P* = 0.51).

### Sensitivity analysis

Most of the RR values did not change substantially following the removal of the included studies one by one. Additionally, the results were stable following a change from the random to the fixed effects models. Notably, when comparing major bleeding associated with TT *versus* DT, the study of Lamberts et al. [48] had a weight of 58.3% in the pooled analysis. After excluding this study, TT was still associated with an increased, although not significant, risk of major bleeding compared with DT (RR = 1.33; 95% CI: 0.85–2.09; *P* = 0.21).

## DISCUSSION

Treatment for AF-related thromboembolism benefits from OAC, whereas antiplatelet therapy is useful for IHD-induced arterial thrombosis. Thus, combination treatment involving OAC and antiplatelet therapy is presumed to prevent the thrombotic complications in patients with AF and IHD [53]. Balancing the risks of bleeding and thrombotic complications is a key consideration that should be carefully considered by clinicians so they can make optimal antithrombotic therapy decisions [54]. To date, the optimal antithrombotic therapy regimen for AF and IHD patients remains a subject of debate. To the best of our knowledge, our meta-analysis is the first to compare the efficacies and safeties of 3 antithrombotic therapy strategies in a large number of patients with AF and IHD. Our principal findings were as follows: (i) compared with DAPT, TT was associated with increased risks of major bleeding, minor bleeding and overall bleeding but did not reduce the risk of death or thrombotic complications (i.e., acute coronary syndrome, stent thrombosis, TE/stroke and MACCEs); and (ii) compared with DT, TT was associated with an increased risk of major bleeding but was not different in terms of the risks of minor bleeding, overall bleeding, death or thrombotic complications. Our results were stable and reliable in the sensitivity analysis.

### TT *versus* DAPT

A previous meta-analysis indicated that TT has no additional beneficial effects in patients undergoing percutaneous coronary interventions (PCIs) compared with DAPT [55]. Another meta-analysis consisting of 14 observational studies also noted that compared with DAPT, TT did not reduce the risk of thrombotic events but did increase the risk of major bleeding in acute coronary syndrome patients [56]. Among patients with AF and IHD, our current study demonstrated similar findings in that TT was equivalent to DAPT in terms of death and thrombotic complications but increased the bleeding events. The increased bleeding risk associated with both OAC agents and antiplatelet agents may be related to the duration of therapy. A study performed by Olson *et al.* reported that the prevalence of major bleeding associated with TT is 2.6–4.6% at 30 days after treatment initiation but increases to 7.4–10.3% at 12 months [57]. Even short-term TT treatment, which has no safe therapeutic window in stented patients with AF, is hazardous with respect to the risk of bleeding [38]. Although the duration of TT may influence the bleeding rates, we could not perform the subgroup analysis because our included studies had different durations of follow-up that varied from 12 months to 74.4 months.

Various clinical risk factors could be associated with an increased risk of bleeding, such as advanced age, uncontrolled hypertension, ischemic heart disease, cerebrovascular disease, labile international normalized ratio (INR) control and previous bleeding episodes [58]. Bleeding risk schemes such as the HAS-BLED (Hypertension, Abnormal renal/liver function, Stroke, Bleeding history or predisposition, Labile INR, Elderly [older than 65 years of age], Drugs/alcohol concomitantly) score contain almost all of the relevant modifiable and partially non-modifiable clinical risk factors for bleeding. The HAS-BLED score should be appropriately used to evaluate patients who are potentially at risk for bleeding and to address the potentially reversible bleeding risk factors. The HAS-BLED score has also been validated in patients with acute coronary syndrome undergoing PCI and in patients on TT [25]. When clinicians tightly control the INR such that it is maintained between 2.0 and 2.5 in AF and IHD patients, TT may be associated with fewer bleeding complications [59].

### TT versus DT

In patients undergoing PCI, there were no differences in the risk of MI, stroke or stent thrombosis between TT and DT [60]. However, this study did not focus on patients with AF. A recently published meta-analysis involving 7,276 anticoagulated patients undergoing PCI indicated that TT confers a higher risk of major bleeding but confers no differences in death, major adverse cardiac events, MI, stent thrombosis, or the thromboembolic event rate compared with DT [61]. Indeed, the WOEST (What is the optimal antiplatelet and anticoagulant therapy in patients with oral anticoagulation and coronary stenting) trial of stented patients receiving anticoagulants found that TT resulted in no differences in composite thromboembolic endpoints (including MI, stroke, stent thrombosis and target-vessel revascularization) but increased the bleeding risk compared to OAC plus clopidogrel [17]. Because these studies included both AF and non-AF anticoagulated patients (*e.g.,* patients with mechanical valves, dilated cardiomyopathy, venous thromboembolisms, and apical aneurysms), our meta-analysis first involved only the AF patients with IHD for further analysis. In accordance with the previous findings by Lamberts *et al.* [38, 39, 48], we demonstrated that TT increased the risk of serious bleeding while eliciting no difference in thrombotic complications. Notably, the increased bleeding risk associated with TT was for minor bleeding in the WOEST trial but for major bleeding in our meta-analysis. Our pooled data from 3 included studies indicated no difference in the risk of minor bleeding between TT and DT, which contrasts with the WOEST trial. Clinicians should not underestimate the effects of minor bleeding because superficial or “nuisance” bleeding may cause the discontinuation of antiplatelet therapy and subsequently result in thrombotic complications [62]. Given the limited number of studies included in our minor bleeding analysis, further studies are warranted to confirm our findings. In contrast to our findings, the WOEST trial indicated a lower risk of death in patients on OAC plus clopidogrel. In the study of Lamberts *et al.* [38], OAC plus clopidogrel was associated with a lower risk of death, whereas OAC plus aspirin was associated with a higher death rate than TT. In our meta-analysis, DT was defined as an oral anticoagulant plus one antiplatelet drug (either clopidogrel or aspirin). Thus, the association might have been attenuated such that TT had a risk of death comparable to that of DT. The discrepancies in patient selection and the antithrombotic regimens of DT between the WOEST trial and our meta-analysis might potentially provide the explanations for differing findings, and further studies are warranted to address this issue.

### Implications and further research

For patients with AF and IHD, the 2014 European revascularization guidelines recommend TT as a priority selection (class IIa, level of evidence C) and recommend DT as an alternative to TT (class IIb, level of evidence B) [63]. The 2016 European Society of Cardiology guidelines recommend DT as an alternative to initial TT to balance the risk of thrombotic complications with the risk of bleeding [64]. However, these guidelines consist mainly of expert consensus opinions that are based mostly on observational studies with small sample sizes. As more attention has been devoted to bleeding events, clinicians and researchers have recommended balancing the risks of thrombotic and bleeding complications when choosing antithrombotic strategies [54] Because there are distinct effects associated with different antithrombotic therapies in patients with AF and IHD, the antithrombotic therapy decisions require a careful, individualized assessment of the benefits and risks of therapy for each patient. Compared with dual therapy, triple therapy yielded more bleeding events but with no difference in the incidence of thrombotic complications. Thus, the benefit of triple therapy seems to have diminished, and the initiation of triple therapy may be unnecessary. Clinicians should be aware of the hazard of adding additional oral anticoagulants to dual therapy.

Although our results provide further support for the previous evidence suggesting that triple therapy is associated with increased risks of major bleeding and with no thromboembolic protection compared with dual therapy, our findings cannot change the approach to current practice. Recently, non-vitamin K antagonist oral anticoagulants (NOACs) have been increasingly used in current practice. NOACs (*e.g.,* dabigatran and rivaroxaban) [65] are associated with lower risks of TE/stroke and intracranial hemorrhage compared to vitamin K antagonists among patients with nonvavular AF. In the ATLAS ACS 2-TIMI 51 (Anti-Xa Therapy to Lower Cardiovascular Events in Addition to Standard Therapy in Subjects with Acute Coronary Syndrome-Thrombolysis in Myocardial Infarction 51) study, the highest dose of rivaroxaban reduced the risk of ischemic events but resulted in excessive bleeding events, whereas very-low-dose rivaroxaban did not increase fatal bleeding events among patients with acute coronary syndrome [66]. Based on these findings, whether the addition of NOACs to dual therapy could be a safer choice is a subject of continued interest. Indeed, in the recent open-label, randomized, controlled, multicenter study (PIONEER AF-PCI) involving 2124 stented patients with AF, both low-dose rivaroxaban (15 mg once daily) plus a P2Y12 inhibitor and very-low-dose rivaroxaban (2.5 mg twice daily) plus DAPT were associated with lower bleeding risks than standard triple therapy with a vitamin K antagonist plus DAPT. The risks of cardiovascular death, MI, and stroke were similar in these 3 groups [67]. Additionally, subsequent randomized trials, such as the RT-AF (Rivaroxaban and Ticagrelor in Atrial Fibrillation) [68], REDUAL-PCI (Randomized Evaluation of Dual Therapy with Dabigatran versus Triple Therapy Strategy with Warfarin in Patients with nonvalvular atrial fibrillation that have undergone percutaneous coronary intervention with Stenting) [69] and AUGUSTUS (Apixaban in NonValvular Atrial Fibrillation with a Recent Acute Coronary Syndrome or Undergoing Percutaneous Coronary Intervention) [70] will provide more data regarding the antithrombotic management of patients with IHD and AF. The inclusion of data on NOACs, when available, would improve future antithrombotic management.

### Limitations

Several potential limitations of this meta-analysis should be carefully addressed. First, the clinical heterogeneity inherent among the included studies could not be resolved. Some of our included studies did not report the outcomes of ischemic stroke and hemorrhagic stroke separately. This aspect is an important issue given the different underlying pathophysiology and differing effects of antithrombotic therapy on these stroke risks. Additionally, almost all the included studies failed to differentiate stented patients undergoing PCI from patients with stable coronary artery disease. Moreover, the definitions employed for outcomes varied among the included studies. Second, the quality of INR control in the warfarin-treated patients was closely related to the outcomes; however, we were unable to conduct this analysis because of a lack of sufficient INR control data. Third, we could not perform a subgroup analysis based on the duration of therapy due to the various follow-up times. Future studies should address this issue because all of the endpoints increase over time due to the duration of treatment. Fourth, the potential role of NOACs as the sole OAC strategy has not been directly assessed due to limited data. In terms of clinical applicability, further studies should be undertaken to investigate the efficacy and safety of NOACs combined with antiplatelet drugs.

## CONCLUSIONS

In summary, our analysis suggests that triple therapy confers an increased hazard of major bleeding with no extra thromboembolic protection compared with dual therapy among patients with AF and IHD. Further randomized studies of NOACs are warranted and may improve antithrombotic management.

## SUPPLEMENTARY MATERIALS FIGURE AND TABLES


