# Supplementary material for: Efficacy and safety of triple versus dual antithrombotic therapy in atrial fibrillation and ischemic heart disease: a systematic review and meta-analysis
Source: Oncotarget. 2017 Sep 14;8(46):81154–66. doi: 10.18632/oncotarget.20870 (PMC5655270; doi:10.18632/oncotarget.20870)
Supplement: Supplementary file 1 [file oncotarget-08-81154-s001.pdf]

Efficacy and safety of triple *versus* dual antithrombotic therapy in atrial fibrillation and ischemic heart disease: a systematic review and meta-analysis

SUPPLEMENTARY MATERIALS

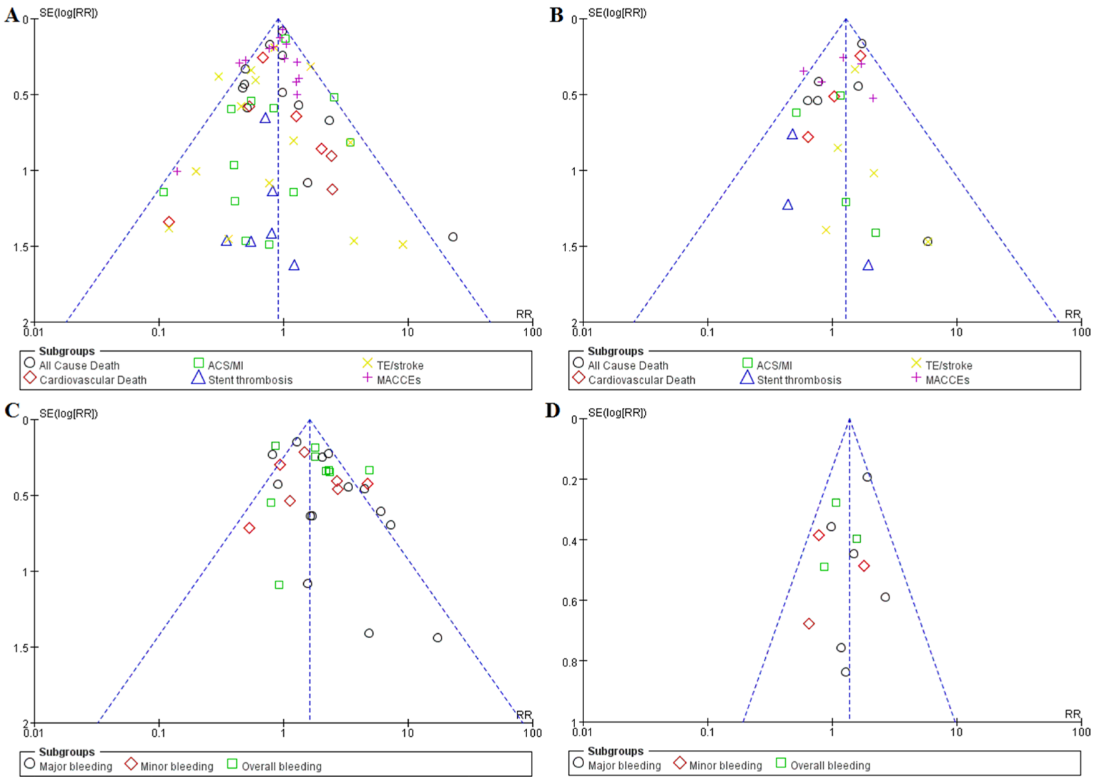

Supplementary Figure 1: Funnel plots of the efficacy and safety outcomes of triple versus dual antithrombotic therapy. (**A**) efficacy of TT versus DAPT; (**B**) efficacy of TT versus DT; (**C**) safety of TT versus DAPT; and (**D**) safety of TT versus DT. Abbreviations: IHD = ischemic heart disease; AF = atrial fibrillation; TT = triple therapy (an oral anticoagulant plus 2 antiplatelet drugs); DAPT = dual therapy (2 antiplatelet drugs); DT = dual therapy (an oral anticoagulant plus one antiplatelet drug); ACS = acute coronary syndrome; TE = thromboembolism; MI = myocardial infarction; MACCEs = major adverse cardiac and cerebrovascular events; RR = relative risk; SE = standard error.

Supplementary Table 1: Electronic search strategies determined on August 2017

Search	Query
**The Cochrane library**	
#1	Coronary Disease OR coronary heart disease ORcoronary artery disease OR drug eluting stents OR percutaneous coronary intervention OR myocardial ischemia OR ischemic heart disease OR acute coronary syndromes OR angina pectoris
#2	atrial fibrillation
#3	#1 and #2
#4	Antithrombotic OR antiplatelet OR clopidogrel OR ticagrelor OR P2Y12 antagonist OR thienopyridine OR prasugrel OR cilostazol OR aspirin OR anticoagulant OR warfarin OR coumarins OR vitamin K antagonists
#5	#3 and #4
#6	Death OR mortality OR all-cause mortality OR cardiovascular mortality OR myocardial infarction OR stroke OR thromboembolism OR cerebrovascular accident OR hemorrhage OR bleeding OR major bleeding OR serious bleeding OR minor bleeding
#7	#5 and #6
#8	#7 and human
**PubMed**	
#1	Coronary Disease[Mesh] OR coronary disease[Text Word] OR coronary heart disease[Text Word] OR coronary artery disease[Mesh] OR coronary artery disease[Text Word] OR drug eluting stents [Mesh] OR drug eluting stents[Text Word] OR percutaneous coronary intervention[Mesh] OR percutaneous coronary intervention [Text Word] OR myocardial ischemia[Mesh] OR myocardial ischemia [Text Word] OR ischemic heart disease[Text Word] OR acute coronary syndromes[Mesh] OR acute coronary syndromes[Text Word] OR angina pectoris[Mesh] angina pectoris[Text Word]
#2	Atrial fibrillation[Mesh] OR Atrial fibrillation[Text Word]
#3	#1 and #2
#4	Antithrombotic[Mesh] OR antithrombotic[Text Word] OR antiplatelet [Mesh] OR antiplatelet[Text Word] OR clopidogrel[Mesh] OR clopidogrel[Text Word] OR ticagrelor [Mesh] OR ticagrelor[Text Word] OR P2Y12 antagonist [Mesh] OR P2Y12 antagonist[Text Word] OR thienopyridine[Mesh] OR thienopyridine [Mesh] OR prasugrel[Mesh] OR prasugrel[Text Word] OR cilostazol[Mesh] OR cilostazol[Text Word] OR aspirin [Mesh] OR aspirin[Text Word] anticoagulant [Mesh] OR anticoagulant [Text Word] warfarin [Mesh] OR warfarin[Text Word] coumarins[Mesh] OR coumarins[Text Word] vitamin K antagonists[Mesh] OR vitamin K antagonists [Text Word]
#5	#3 and #4
#6	Death[Mesh] OR death [Text Word] OR mortality [Mesh] OR mortality[Text Word] OR all-cause mortality [Mesh] OR all-cause mortality[Text Word] cardiovascular mortality [Mesh] OR cardiovascular mortality[Text Word] OR myocardial infarction [Mesh] OR myocardial infarction [Text Word] OR stroke [Mesh] OR stroke[Text Word]OR thromboembolism [Mesh] OR thromboembolism [Text Word] cerebrovascular accident [Mesh] OR cerebrovascular accident[Text Word] OR hemorrhage [Mesh] OR hemorrhage [Text Word] OR bleeding [Mesh] OR bleeding[Text Word] OR major bleeding [Mesh] OR major bleeding [Text Word] serious bleeding [Text Word]OR minor bleeding [Mesh] OR minor bleeding [Text Word]
#7	#5 and #6
#8	#7 and human[MeSH]
**EMBASE**	
#1	‘ Coronary Disease’/exp OR ‘ coronary heart disease ‘/exp OR ‘coronary artery disease ‘/exp OR ‘ drug eluting stents ‘/exp OR ‘ percutaneous coronary intervention ‘/exp OR myocardial ischemia ‘/exp OR ‘ ischemic heart disease ‘/exp OR ‘ acute coronary syndromes ‘/exp OR ‘ angina pectoris ‘/exp
#2	‘atrial fibrillation’/exp
#3	#1 and #2
#4	‘Antithrombotic’/exp OR ‘ antiplatelet ‘/exp OR ‘ clopidogrel ‘/exp OR ‘ ticagrelor ‘/exp OR ‘ P2Y12 antagonist ‘/exp OR thienopyridine ‘/exp OR ‘ prasugrel ‘/exp OR ‘ cilostazol ‘/exp OR ‘ aspirin ‘/exp OR ‘ anticoagulant ‘/exp OR ‘ warfarin ‘/exp OR ‘ coumarins ‘/exp OR ‘ vitamin K antagonists ‘/exp
#5	#3 and #4
#6	‘ Death ‘/exp OR ‘ mortality ‘/exp OR ‘ all-cause mortality ‘/exp OR ‘ cardiovascular mortality ‘/exp OR ‘ myocardial infarction ‘/exp OR ‘stroke ‘/exp OR ‘ thromboembolism ‘/exp OR ‘ cerebrovascular accident ‘/exp OR ‘ hemorrhage ‘/exp OR ‘ bleeding ‘/exp OR ‘ major bleeding ‘/exp OR ‘ serious bleeding ‘/exp OR ‘ minor bleeding ‘/exp
#7	#5 and #6
#8	#7 and ‘human’/de

Supplementary Table 2: Definitions of safety and efficacy outcomes in the 17 included studies

References (first author, year)	ACS§	TE/stroke※	MACCEs	Stent thrombosis	Bleeding*
Choi, 2017	nonfatal MI	ischemic or hemorrhagic stroke	cardiovascular death, MI, or stroke	the Academic Research Consortium classification[1]	TIMI[2]
De Vecchis, 2016	unstable angina and nonfatal MI[3]	deep vein thrombosis,pulmonary embolism,stroke/TIA	MI, TVR and Stroke	NA	NA
Fang, 2016	any troponin elevation or suggestive symptoms detected	NA	NA	NA	GUSTO
Lopes, 2016	NA	NA	stroke, TIA, MI, non-CNS embolism or revascularization	NA	ISTH[4]
Sambola, 2016	acute MI[5]	systemic embolism, ischemic or hemorrhagic stroke	all-cause mortality ,MI, stent thrombosis or TVR	the Academic Research Consortium[1]	TIMI[6] and PRISM-PLUS[7]
Kawai, 2015	nonfatal MI	ischemic or hemorrhagic stroke	all-cause death, nonfatal MI, intracranial bleeding, and cerebral infarction	NA	TIMI[6]
Mennuni, 2015	NA	NA	all-cause mortality, nonfatal MI and stroke	NA	BARC[8]
Hess,2015	MI	ischemic stroke	all-cause mortality, MI and Stroke	NA	International Classification of Diseases Ninth Revision codes
Kang, 2015	nonfatal MI	ischemic cerebrovascular accident	all-cause mortality, MI, TVR and ischemic Stroke	NA	Global Utilization of Streptokinase and Tissue Plasminogen Activator for Occluded Coronary Arteries criteria[9]
Lamberts, 2014	NA	ischemic stroke and systemic arterial embolism	NA	NA	ISTH[4]
Rubboli, 2014	MI[3]	stroke/transient ischemic attack, systemic embolism	stroke, Peripheral embolism, MI, TVR and stent thrombosis	Academic Research Consortium classification and included definite and probable events[10]	BARC[8]
Suh, 2013	MI	ischemic stroke	cardiac death, MI, TVR, and stroke	the Academic Research Consortium	NA
Dabrowska, 2013	recurrent ACS	thromboembolic events	NA	NA	bleeding requiring hospitalization and/ or discontinuation of any antithrombotic medication
Caballero, 2013	MI	NA	all-cause mortality, MI and TVR	NA	PRISM-PLUS[7]
Fosbol, 2013	International Classification of Diseases, Ninth Revision (ICD-9), diagnosis codes	International Classification of Diseases, Ninth Revision (ICD-9), diagnosis codes	all-cause mortality, MI and stroke	International Classification of Diseases, Ninth Revision (ICD-9), diagnosis codes	International Classification of Diseases, Ninth Revision (ICD-9), diagnosis codes
Gao, 2010	MI	ischemic stroke	all-cause mortality, MI, TVR, Stent thrombosis and Stroke	reference[11]	TIMI[12]
Maegdefessel, 2008	MI	ischemic stroke	NA	NA	Severe gastrointestinal bleedings requiring endoscopic interventions and at least three blood transfusions

§based on the universal definition of MI.※by a neurologist and confirmed by computed tomography/magnetic resonance imaging. *overall bleeding is equal to major bleeding plus minor bleeding.

Abbreviations: NA = not available; ACS = Acute coronary syndrome; MACCEs=major adverse cardiac and cerebral events; TE = thromboembolism; MI = myocardial infarction; TVR = target vessel revascularisation; TIMI=Thrombolysis In Myocardial Infarction criteria; PRISM-PLUS = Platelet Receptor Inhibition in Ischemic Syndrome Management in Patients Limited by Unstable Signs and Symptoms; TIA = transient ischemic attack; GUSTO=Global Use of Strategies to Open Coronary Arteries; ISTH = International Society of Thrombosis and Haemostasis; BARC=Bleeding Academic Research Consortium.

REFERENCES

1. Laskey WK, Yancy CW, Maisel WH. Thrombosis in coronary drug-eluting stents: report from the meeting of the Circulatory System Medical Devices Advisory Panel of the Food and Drug Administration Center for Devices and Radiologic Health, December 7-8, 2006. Circulation. 2007; 115:2352–2357.
2. Wiviott SD, Antman EM, Gibson CM, Montalescot G, Riesmeyer J, Weerakkody G, Winters KJ, Warmke JW, McCabe CH, Braunwald E. Evaluation of prasugrel compared with clopidogrel in patients with acute coronary syndromes: design and rationale for the TRial to assess Improvement in Therapeutic Outcomes by optimizing platelet InhibitioN with prasugrel Thrombolysis In Myocardial Infarction 38 (TRITON-TIMI 38). Am Heart J. 2006; 152:627–635.
3. Thygesen K, Alpert JS, White HD. Universal definition of myocardial infarction. J Am Coll Cardiol. 2007; 50:2173–2195.
4. Schulman S, Kearon C. Definition of major bleeding in clinical investigations of antihemostatic medicinal products in non-surgical patients. J Thromb Haemost. 2005; 3:692–694.
5. Lip GY, Windecker S, Huber K, Kirchhof P, Marin F, Ten BJ, Haeusler KG, Boriani G, Capodanno D, Gilard M, Zeymer U, Lane D, Storey RF, et al. Management of antithrombotic therapy in atrial fibrillation patients presenting with acute coronary syndrome and/or undergoing percutaneous coronary or valve interventions: a joint consensus document of the European Society of Cardiology Working Group on Thrombosis, European Heart Rhythm Association (EHRA), European Association of Percutaneous Cardiovascular Interventions (EAPCI) and European Association of Acute Cardiac Care (ACCA) endorsed by the Heart Rhythm Society (HRS) and Asia-Pacific Heart Rhythm Society (APHRS). Eur Heart J. 2014; 35:3155–3179.
6. Chesebro JH, Knatterud G, Roberts R, Borer J, Cohen LS, Dalen J, Dodge HT, Francis CK, Hillis D, Ludbrook P, Et A. Thrombolysis in Myocardial Infarction (TIMI) Trial, Phase I: A comparison between intravenous tissue plasminogen activator and intravenous streptokinase. Clinical findings through hospital discharge. Circulation. 1987; 76:142–154.
7. Inhibition of the platelet glycoprotein IIb/IIIa receptor with tirofiban in unstable angina and non-Q-wave myocardial infarction. N Engl J Med. 1998; 338:1488–1497.
8. Mehran R, Rao SV, Bhatt DL, Gibson CM, Caixeta A, Eikelboom J, Kaul S, Wiviott SD, Menon V, Nikolsky E, Serebruany V, Valgimigli M, Vranckx P, et al. Standardized bleeding definitions for cardiovascular clinical trials: a consensus report from the Bleeding Academic Research Consortium. Circulation. 2011; 123:2736–2747.
9. An international randomized trial comparing four thrombolytic strategies for acute myocardial infarction. N Engl J Med. 1993; 329:673–682.
10. Cutlip DE, Windecker S, Mehran R, Boam A, Cohen DJ, van Es GA, Steg PG, Morel MA, Mauri L, Vranckx P, McFadden E, Lansky A, Hamon M, et al. Clinical end points in coronary stent trials: a case for standardized definitions. Circulation. 2007; 115:2344–2351.
11. Mauri L, Hsieh WH, Massaro JM, Ho KK, D'Agostino R, Cutlip DE. Stent thrombosis in randomized clinical trials of drug-eluting stents. N Engl J Med. 2007; 356:1020–1029.
12. Rao AK, Pratt C, Berke A, Jaffe A, Ockene I, Schreiber TL, Bell WR, Knatterud G, Robertson TL, Terrin ML. Thrombolysis in Myocardial Infarction (TIMI) Trial--phase I: hemorrhagic manifestations and changes in plasma fibrinogen and the fibrinolytic system in patients treated with recombinant tissue plasminogen activator and streptokinase. J Am Coll Cardiol. 1988; 11:1–11.

Supplementary Table 3: Quality assessment of the 17 included studies

References (first author, year)	Selection				Comparability	Outcome			Total
	Exposed cohort	Non-exposed cohort	Ascertainment of exposure	Outcome of interest		Assessment of outcome	Length of follow-up	Adequacy of follow up	
De Vecchis, 2016[44]	*	*	*			*	*	*	6
Lopes, 2016[39]	*	*	*		**	*	*	*	8
Fan, 2016[45]	*	*	*			*	*	*	6
Sambola, 2016[42]	*	*	*			*	*	*	6
Kawai, 2015[8]	*	*	*		*	*	*	*	7
Mennuni, 2015[40]	*	*	*		**	*	*	*	8
Hess,2015[38]	*	*	*		*	*	*	*	7
Kang, 2015[7]	*	*	*		**	*	*	*	8
Lamberts, 2014[11]	*	*	*		*	*	*	*	7
Rubboli, 2014[41]	*	*	*		**	*	*	*	8
Caballero, 2013[5]	*	*	*			*	*	*	6
Suh, 2013[47]	*	*	*			*	*	*	6
Dabrowska, 2013[37]	*	*	*			*	*	*	6
Fosbol, 2013[44]	*	*	*		**	*	*	*	8
Gao, 2010[6]	*	*	*			*	*	*	6
Maegdefessel, 2008[46]	*	*	*			*	*	*	6
Choi, 2017[43]	*	*	*		**	*	*	*	8

Supplementary Table 4: General characteristics of the 17 studies included in this meta-analysis.
See Supplementary_Table_4
